# Transcriptome analysis reveals crucial genes involved in the biosynthesis of nervonic acid in woody *Malania oleifera* oilseeds

**DOI:** 10.1186/s12870-018-1463-6

**Published:** 2018-10-19

**Authors:** Tianquan Yang, Qian Yu, Wei Xu, De-zhu Li, Fu Chen, Aizhong Liu

**Affiliations:** 1 Germplasm Bank of Wild Species, Kunming Institute of Botany, Chinese Academy of Sciences, 132 Lanhei Road, Kunming, 650204 China; 2 Key Laboratory for Forest Resources Conservation and Utilization in the Southwest Mountains of China, Ministry of Education, Southwest Forestry University, Kunming, 650224 China; 3 Department of Economic Plants and Biotechnology, and Yunnan Key Laboratory for Wild Plant Resources, Kunming Institute of Botany, Chinese Academy of Sciences, 132 Lanhei Road, Kunming, 650204 China; 4 The Camellia Institute, Yunnan Academy of Forestry, Kunming, China; 5 University of Chinese Academy of Sciences, Beijing, China

**Keywords:** Nervonic acids, *Malania oleifera*, Transcriptome, Oilseed, Gene expression, Oil biosynthesis

## Abstract

**Background:**

*Malania oleifera* Chun et Lee (Olacaceae), an evergreen broad-leaved woody tree native to southwest China, is an important oilseed tree. Its seed oil has a high level of nervonic acid (cis-tetracos-15-enoic acid, over 60%), which is essential for human health. *M. oleifera* seed oil is a promising source of nervonic acid, but little is known about the physiological and molecular mechanisms underlying its biosynthesis.

**Results:**

In this study, we recorded oil accumulation at four stages of seed development. Using a high-throughput RNA-sequencing technique, we obtained 55,843 unigenes, of which 29,176 unigenes were functionally annotated. By comparison, 22,833 unigenes had a two-fold or greater expression at the fast oil accumulation stage than at the initial stage. Of these, 198 unigenes were identified as being functionally involved in diverse lipid metabolism processes (including de novo fatty acid synthesis, carbon chain elongation and modification, and triacylglycerol assembly). Key genes (encoding KCS, KCR, HCD and ECR), putatively responsible for nervonic acid biosynthesis, were isolated and their expression profiles during seed development were confirmed by quantitative real-time PCR analysis. Also, we isolated regulatory factors (such as WRI1, ABI3 and FUS3) that are putatively involved in the regulation of oil biosynthesis and seed development.

**Conclusion:**

Our results provide novel data on the physiological and molecular mechanisms of nervonic acid biosynthesis and oil accumulation in *M. oleifera* seeds, and will also serve as a starting point for biotechnological genetic engineering for the production of nervonic acid resources.

**Electronic supplementary material:**

The online version of this article (10.1186/s12870-018-1463-6) contains supplementary material, which is available to authorized users.

## Background

Exploration and utilization of non-timber biological resources from woody trees has long been an important area of forestry research. Oilseeds derived from woody trees have great potential to meet the increasing demand for vegetable oils for food or industrial usage. In particular, oilseed trees that produce unusual fatty acids (FAs) provide critical woody non-timber sources of unique FAs. *Malania oleifera* Chun et Lee (2n = 26) [1], a monotypic species belonging to the family Olacaceae, is an evergreen broad-leaved woody tree native to southwest China [2], mainly distributed in limited regions of Guangxi and Yunnan province [3, 4]. For many years, the seeds of *M. oleifera* have been used for making edible oils and consumed by local people. Seed oil of *M. oleifera* is distinctive for its high level of 15c-tetracosenoic acid (C24:1Δ15), a kind of Very Long-Chain Monounsaturated Fatty Acid (VLCMFA), namely nervonic acids (over 60% of total fatty acids).

Nervonic acids were first discovered in the sphingolipid of sea animals (such as sharks). They are chiefly found in nervous and brain tissues, comprising the white matter of animal brains and myelinated nerve fibers [5, 6]. Altered nervonic acid levels in human blood or tissues can cause a variety of diseases. They are implicated in a number of neurological disorders and in some mental illnesses, including schizophrenia, psychosis and attention deficit disorder. Nervonic acid oils have become important targets for pharmaceutical and nutraceutical applications in the prevention and treatment of neurological disorders and associated diseases, including multiple sclerosis, adrenoleukodystrophy, Zeellweger syndrome and Alzhemier’s disease [6–13]. Notably, there has been some evidence that nervonic acid inhibits the human immunodeficiency virus-1 (HIV-1) reverse transcriptase in a dose-dependent manner [14]. Thus, nervonic acid is a strong candidate for further evaluation as a bioactive lipid supplement for the promotion of human health. However, the availability of nervonic acid is currently limited because sea animal sources are insufficient to meet the growing market demand for nervonic acid.

There is an urgent need for a sustainable source of nervonic acids derived from plant oils. Recently, several plant seeds including *Lunaria annua* (honesty), *Borago officinalis* (borage), *Cannabis sativa* (hemp), *Acer truncatum* (purple blow maple), *Tropaeolum speciosum* (flame flower), *Cardamine graeca* (bittercress) and *Malania oleifera* (garlic-like fruit) were found to contain nervonic acid within storage lipids in the form of triacylglycerol (TAG) [15–18]. Although the nervonic acid content in *Acer truncatum* and *Lunaria annua* seeds are low (5% and 20% respectively) these two plants have been considered to be potentially important resources for developing nervonic acid products [16, 18]. The high market demand for nervonic acid incentivizes the development of a refined, nervonic acid-enriched plant oil. *M. oleifera* is a good candidate for the discovery and development of nervonic acid resources because of its nervonic acid-enriched seed oils, but the physiological and molecular mechanisms underlying the biosynthesis of nervonic acid-enriched oils in *M. oleifera* seeds remain unknown.

Two main pathways are involved in oil accumulation in plant seeds: fatty acid (FA) de novo synthesis (including FAs carbon chain elongation and desaturation) and TAG assembly. FA biosynthesis mainly takes place within plastids and is initiated by the irreversible carboxylation of acetyl-CoA to form malonyl-CoA by acetyl-CoA carboxylase (ACCase). The malonyl group is transferred to ACP (Acyl-carrier protein). Next, fatty acid synthase (FAS) catalyzes the conversion of acetyl-CoA and malnoyl-ACP to 16:0 and 18:0 acyl-ACP. FAS is a protein complex consisting of several individual enzymes, including a set of β-ketoacyl-ACP synthases (KASs) that are enzymes for FA biosynthesis [19, 20]. In addition, 18:0-ACP can be desaturated to 18:1-ACP by stearoyl-ACP desaturase (SAD), which determines the level of unsaturated FAs (UFAs) in the plant cell. After that, these fatty acyl-ACP chains are converted into acyl-CoAs and transferred to endoplasmic reticulum (ER) for further elongation, desaturation and modification, which generates a variety of FAs like very-long-chain fatty acids (VLCFAs) and polyunsaturated FAs (PUFAs). Based on the initial carbon chain backbone of C16:0, biosynthesis of nervonic acids (C24:1Δ15) is considered to comprise the sequential addition of two carbons by four successive enzymatic reactions gathered by a enzymatic complex [21–23]. The first step is catalysis by membrane-bound 3-ketoacyl-CoA synthase (KCS or FA elongase, FAE), which is a key gene for FA elongation in ER [24–28]. The resulting 3-ketoacyl-CoA is then reduced by a 3-ketoacyl-CoA reductase (KCR) generating a 3-hydroxy-acyl-CoA [29, 30]. The third step is dehydration by the reaction of 3-hydroxacyl-CoA dehydratase (HCD, also known as PASTICCINO 2, or PAS2) to a trans-2,3-enoyl-CoA [31], which is finally reduced by the trans-2,3-enoyl-CoA reductase (ECR) to yield a two-carbon elongated acyl-CoA [32]. Nervonic acid was synthesized by these four enzymatic reactions after three cycles, using mono-unsaturated fatty acids (MUFAs) 18:1 as the substrate. Finally, the TAG assembly consumes acyl-CoAs using substrate glycerol 3-phosphate with four consecutive enzymes that sequentially transfer acyl-CoAs to sn-1, − 2, − 3 positions in glycerol 3-phosphate in the ER, including Glycerol-3-phosphate acyltransferase (GPAT), Lysophosphlipid acyltransferase (LPAT), Phosphatidic acid phosphatase (PAP) and Diacylglycerol acyltransferase (DGAT) [33, 34].

In this study, we de novo assembled and characterized the transcriptome of *M. oleifera* seeds at two developmental stages*.* A number of unigenes involved in the processes of FA biosynthesis (in particular, carbon chain elongation) and TAG assembly were identified. To our knowledge, this study is the first report on characterizing the transcriptome data in woody oilseeds which produce rich nervonic acid oils. These transcripts identified in *M. oleifera* seeds provide valuable resources for discovering novel genes responsible for the biosynthesis of nervonic acid oils in plants.

## Methods

### Plant materials and determination of oil accumulation

**Fig. 1:**
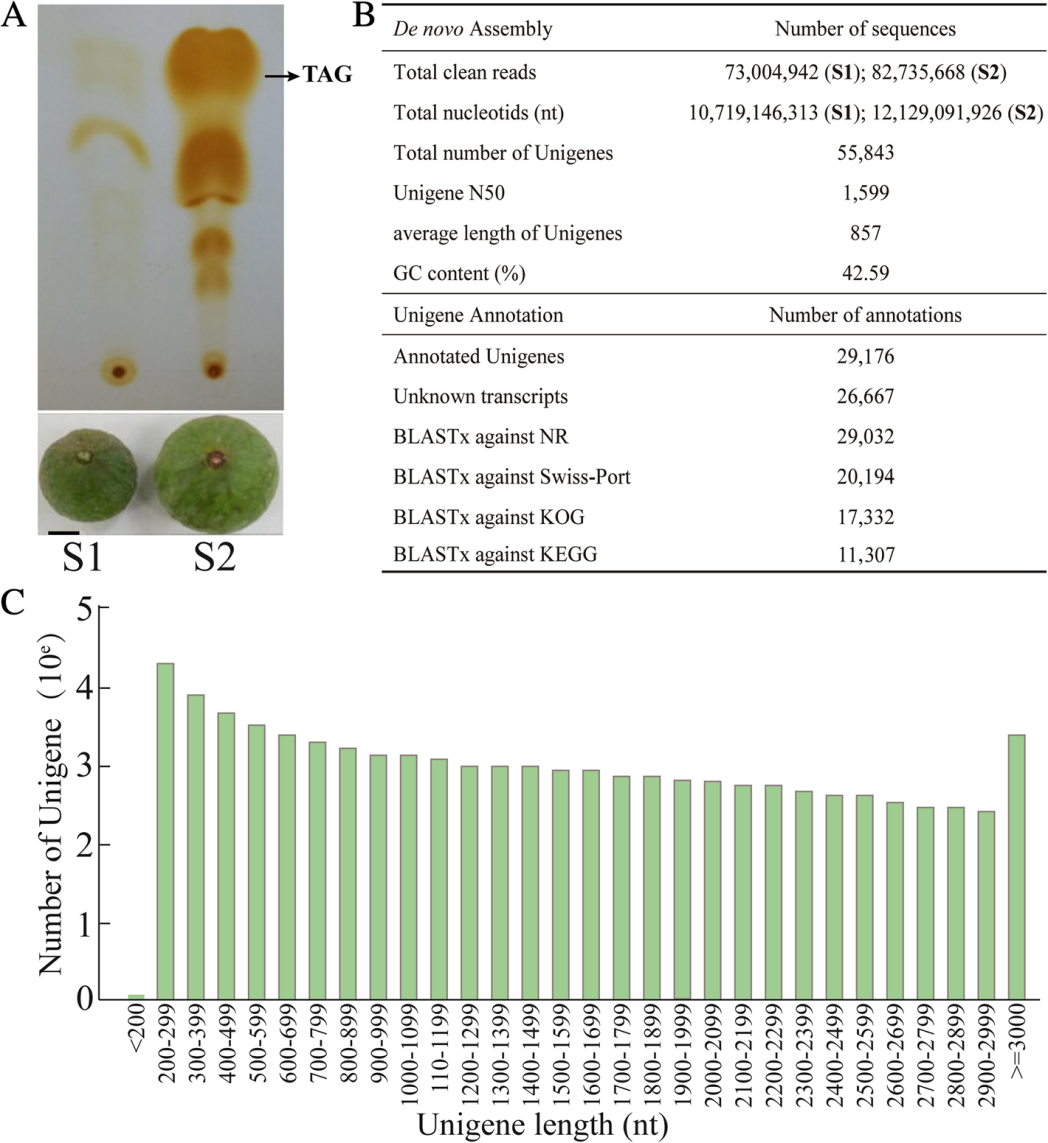
The seeds at two different developmental stages and analysis of transcriptome sequencing. **a** Thin layer chromatography analysis of triacylglycerols from *M. oleifera* seeds at S1 and S2 stage, scale bar = 1.0 cm. **b** Summary of sequencing data, assembly and annotation. **c** Distribution of sequence length of unigenes

Samples were collected from wild *M. oleifera* trees growing in Guangnan county (voucher No. 0814046, identified by Li-gong Lei and deposited at KUN), Yunnan, China under natural climate conditions. The collection of all samples completely complies with local and national legislation permission. We observed the development process of *M. oleifera* seeds from female flowers pollinated to mature seeds. Mature female flowers were tagged when the stigma was fully expanded. The young leaves, tender stems and developing seeds at four stages were collected. Three biological replicates were collected for each tissue type. Samples were immediately frozen in liquid nitrogen, and subsequently stored at − 80°Cfor subsequent RNA isolation. To investigate nervonic acid content during seed development, total lipids were extracted from seeds at each stage of development, using the hexane/isopropanol (3:2, *v*/v) method. Total lipids were dissolved in hexane and the neutral lipids were separated by one-dimensional TLC, as described in our previous study [35]. Fatty acid methyl esters (FAMEs) were prepared from the fatty acids of total lipids as previously described and were determined by Gas Chromatography-Mass Spectrometry [36]. The seeds from the initial oil accumulation stage (S1) and the fast oil accumulation stage (S2) were selected for transcriptome sequencing (see Fig. 1a).

### Transcriptome sequencing for developing seeds

For two stages (the initial and fast oil accumulation stages) of developing seeds, three independent samples collected from different fruits were pooled equally to generate three biological replicates. Total RNA was isolated using RNAprep pure Plant Kit (TIANGEN, DP432), following the manufacturer’s protocols. For each sample, High-quality RNA was enriched by Oligo (dT) beads. The enriched mRNA was fragmented into short fragments and reverse transcripted into cDNA with random primers. The cDNA fragments were purified with QiaQuick PCR extraction kit, end-repaired and ligated to Illumina sequencing adapters. The ligation products were selected according to their size by agarose gel electrophoresis, and initially amplified by PCR. The PCR production was constructed into a cDNA library and sequenced on Illumina HiSeq4000™ system in BGI-Shenzhen.

### Transcriptome analysis

After sequencing, the raw reads were preprocessed to filter out clipped adapter sequences, low-quality reads (Q value ≤20 or containing ambiguous nucleotides) and contaminated sequences. The clean reads were subjected to de novo assembly using the Trinity, a short reads assembling program [37]. Based on the overlap of assembled contigs, the fragments were merged or extended into much longer transcripts to form a set of non-redundant unigenes. To further obtain these unigenes’ function, we employed BLASTX (e-value < 0.00001) to search the public databases with the following order: NCBI non-redundant (Nr), Swiss-Prot, KEGG, and COG/KOG. Meanwhile, GO annotation of unigenes was performed by Blast2GO software [38]. GO classification and enrichment analysis of unigenes was performed using WEGO software [39].

The expression level of unigenes was counted and normalized by RPKM (Reads Per Kb per Million reads) [40]. The formula of RKPM is as follows: RPKM = (1000000*C)/(N*L/1000), where C represents the number of reads uniquely mapping to Unigene; L represents the length (base number) of the Unigene; and N represents the number of total reads uniquely mapped to all Unigenes. Unigenes with significantly different expression were determined by FDR ≤ 0.001 (false discovery rate that was used to rectify the *p*-value for multiple testing) and fold-change ≥2 in two samples.

### Validation of full-length cDNA and expression level

Based on the presence of 5′ and 3′ untranslated sequences, the full-length cDNAs of unigenes potentially involved in nervonic acid biosynthesis were isolated. Subsequently, they were further confirmed by RT-PCR and sequencing. The expression profiles of unigenes were carried out in different tissues. The young leaf, tender stems, and seeds from two developmental stages were subjected to quantitative real-time PCR (qRT-PCR). Total RNAs were isolated (mentioned above) and reverse transcripted using PrimerScrip™ RT reagent Kit with gDNA Erases (Takara, China). qRT-PCR was performed on the CFX96 machine (Bio-Rad, USA) according to the following program: precycling steps of 95 °C for 2 min then 40 cycles of 95 °C for 30 s, 56 °C for 30 s, and 72 °C for 30 s. The UBE (ubiquitin-conjugating enzyme) gene, Unigene0016233 of *M. oleifera*, was used as an internal reference to normalize the relative expression level of all genes. All primers used in this study were listed in Table S1 (see Additional file 1).

## Results

### Transcriptome sequencing and de novo assembly

To investigate nervonic acid accumulation during seed development in *M. oleifera*, we collected seeds at four stages of development (named as S1-S4), as determined by their seed size, and analyzed the fatty acid species for each sample. As seeds developed, the nervonic content increased gradually, from 0.88% (S1) to 63.79% (S4) of the total fatty acids (Additional file 2). Thin layer chromatography analysis showed that while fruits are young (S1) there is an initial stage of oil accumulation. During the expansion growth period (S2) there is a rapid oil accumulation (Fig. 1a). Therefore, we selected these two stages (the initial and fast oil accumulation stages) of seed development for transcriptome sequencing.

Two cDNA libraries were constructed from two stages of developing seeds (Fig. 1a) and yielded a total of 22.8 gigabases (Gb) nucleotides by Illumina high-throughput sequencing. After strict reads filtering, we obtained about 73 and 82 million 150-bp paired-end reads from S1 and S2, respectively. The Trinity package was employed to assemble all high-quality reads to generate a reference transcriptome. As a result, we obtained 55,843 non-redundant unigenes with an average sequence length of 857 bp and an N50 of 1,599 bp (Fig. 1b). The average GC content of *M. oleifera* unigenes was 42.59%. The size distribution showed that 15,304 unigenes (27.41%) were longer than 1 kb (Fig. 1c).

### Functional annotation of non-redundant unigenes of *Malania oleifera*

**Fig. 2:**
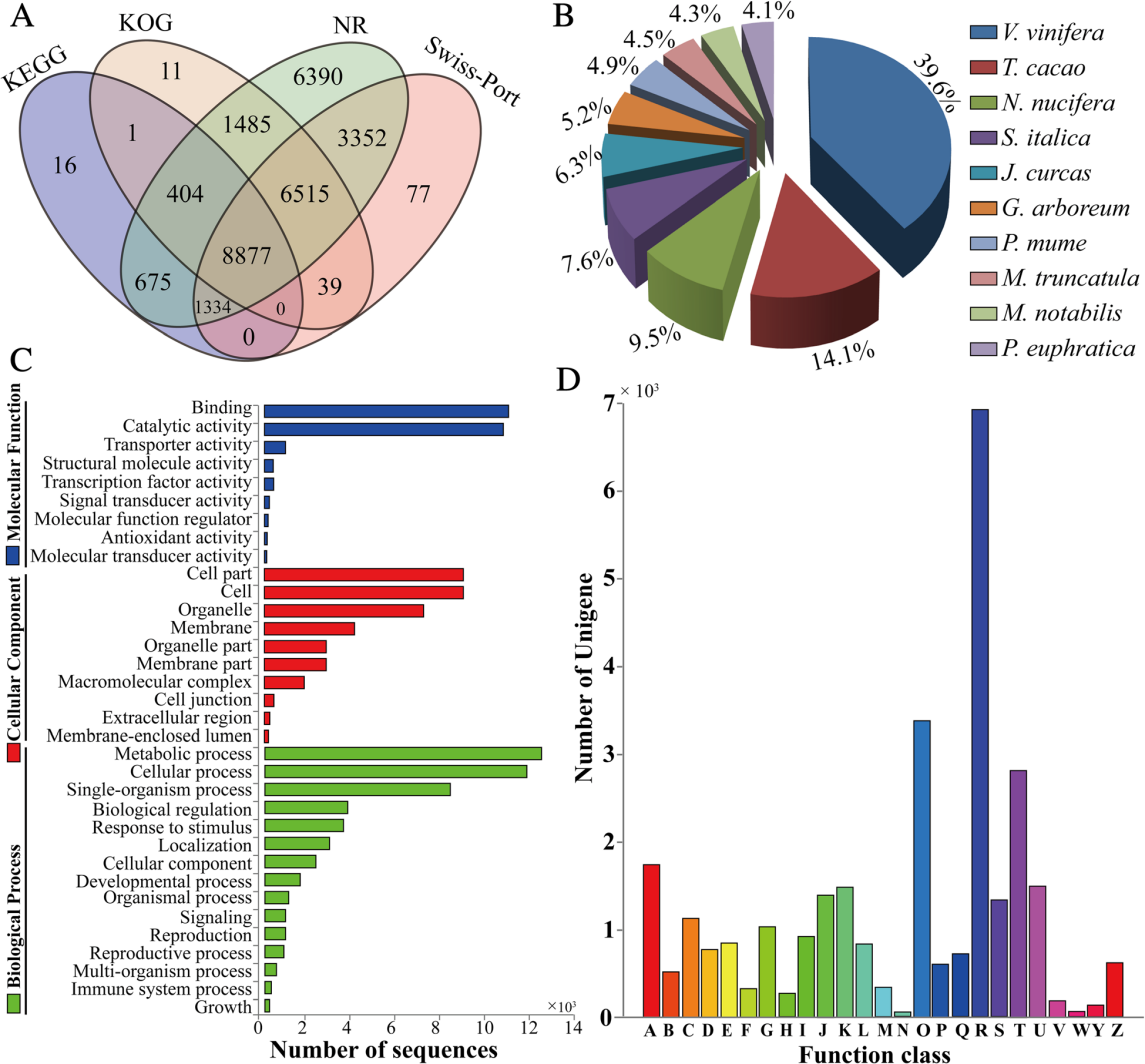
Functional annotation and classification of unigenes. **a** The number of annotated unigene in four public database including NR, Swiss-Port, KOG and KEGG. **b** Species distribution for top BLAST hits for each unigene in the Nr database. **c** GO category distribution of transcripts derived from *M. oleifera*. The histogram shows the results of classifying 55,843 genes into three classification of GO terms. **d** Clusters of Orthologous Group (COG) classification of *M.oleifera* unigenes which were classified into 25 functional categories

To uncover the potential function of unigenes in *M. oleifera*, they were compared against the public databases (as mentioned in the materials and methods section) for annotation by using TBLASTX search with an E-value threshold of 10^− 5^. Of 55,843 unigenes, 29,032 (51.99%), 20,194 (36.16%), 17,332 (31.04%) and 11,307 (20.05%) had significant hits in NR, Swissprot, KOG and KEGG, respectively (Figs. 1b and 2a). There were 8,877 unigenes which had significant hits in all of the four databases (Fig. 2a). In total, 29,176 (52.25%) unigenes were annotated and 26,667 (47.75%) remain unknown. As shown in Additional file 3, about 42.0% of unigenes showed very strong homology by BLASTx (E-value < le^− 100^), 37.4% of those had an E-value between 1e^− 50^ and 1e^− 20^ and the remaining 20.6% showed homology (1e^− 20^ < E-value <1e^− 5^) using the NCBI nr database. A similar E-value distribution of the mapped unigenes was found in Swissprot, KOG and KEGG (Additional file 3). Based on NR annotations, BLAST search analysis further revealed that 7,051 (39.6%) had the most similar sequences to proteins from *Vitis vinifera*, followed by *Theobroma cacao*, 2,519 (14.1%), *Nelumbo nucifera*, 1,696 (9.5%), *Setaria italica*, 1,351(7.6%), *Jatropha curcas*, 1,126 (6.3%). The 10 top-hit species containing homologous sequences are shown in Fig. 2b.

### Functional classification of *Malania oleifera* unigenes

Gene Ontology (GO) term system was employed to classify the functions of predicted unigenes. There were 20,423 unigenes that could be annotated to one or more terms under three GO categories including cellular component, biological process, and molecular function (Fig. 2c). In the molecular function division, binding (45.39%) and catalytic activity (44.48%) represented the dominant GO terms. In the cellular component division, GO terms related to cell parts (23.84%) and cell (23.84%) were in joint first place, followed by organelle (19.03%). For the biological processes, the terms related to metabolic processes (24.11%) and cellular processes 11,815 (22.8%) were dominant, followed by single-organism processes 8,352 (16.12%), signaling 957 (1.85%), reproduction 914 (1.76%) and reproductive processes 903 (1.74%). Besides, all unigenes were also subjected to search against the Clusters of Orthologous Groups database (COG). Overall, 17,332 unigenes were clustered into 25 function classes (Fig. 2d). Among these classes, the general function (R, 6,932 hits) represented the largest group (23.34%), followed by posttranslational modification, protein turnover and chaperones (O, 3,395 hits, 11.43%), signal transduction mechanisms (T, 2802 hits, 9.43%). In addition, a small fraction of unigenes were classified into energy production and conversion (C, 1129 hits, 3.80%), lipid transport and metabolism (I, 928 hits, 3.12%) and secondary metabolites biosynthesis, transport and catabolism (Q, 709 hits, 2.39%) (Fig. 2d).

To explore the main pathways in *M. oleifera* seeds, all unigenes were also used to search against the KEGG classification system. We found that these unigenes were classified into 130 KEGG pathways (Additional file 4). Among five main categories, the largest group was pathways related to metabolism (6,217 hits, 60%), followed by genetic information processing (2,787 hits, 27%), cellular processes (538 hits, 5%), environmental information processing (458 hits, 5%), and organismal system (286 hits, 3%). Among these 130 pathways, the maps with the highest unigene representation (467) were ribosome pathway (ko03010), followed by carbon metabolism (408), biosynthesis of amino acids (353) and protein processing in endoplasmic reticulum (321).

### Identification of transcription factors in *M. oleifera* seed

**Fig. 3:**
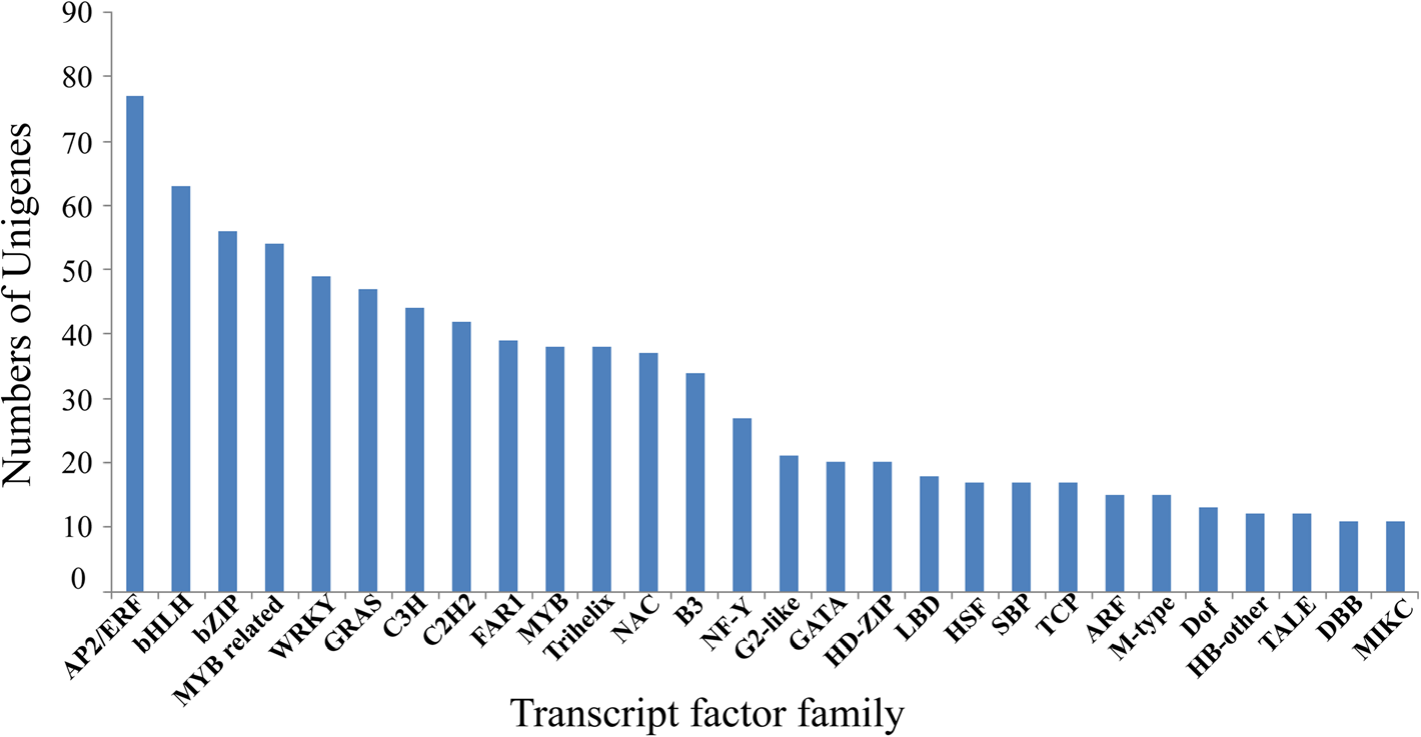
The number distribution of different transcription factor family indentified in Malania oleifera

There is increasing evidence that transcription factors (TFs) play critical roles in regulating the plant growth and development. Here, we identified 978 unigenes encoding the transcription factors belonging to 57 families. Among these TF families, there were 28 families including more than ten members. Of these, the top 6 families are AP2/ERF (77 unigenes), bHLH (63 unigens), bZIP (56 unigenes), MYB (52 unigenes), WRKY (49 unigenes) and GRAS (47 unigenes) which are the largest known TF families in plants (Fig. 3). Identification of these TFs here provides additional understanding of the potential molecular mechanisms of seed development and storage oil accumulation in *M. oleifera*.

### Differentially expressed genes at the two developmental stages

**Fig. 4:**
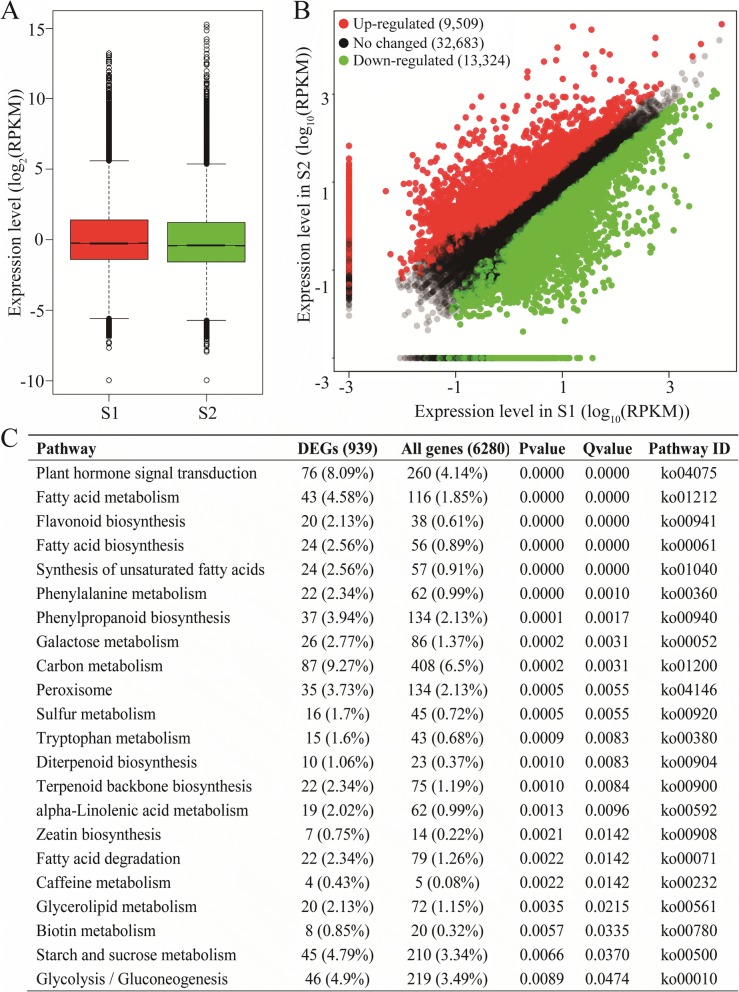
Analysis of differentially expressed unigenes at the two different development stages. **a** Expression level of unigene between the initial stage (S1) and the fast oil accumulation stage (S2). **b** The differentially expressed unigenes at S2 stage relative to S1 stage. The red, black and green dot represent un-regulated, no changed and down-regulated unigenes. **c** KEGG enrichment analysis of differentially expressed unigenes

To identify significant differentially expressed genes in the two stages (the initial and fast oil accumulation stages), the expression levels of all unigenes were normalized to the RPKM value (the reads per kb per million read). Overall, the expression level of unigenes at stage S1 was comparable to that at stage S2 (Fig. 4a). Unigenes with a fold change ≥2 and a false discovery rate (FDR) < 0.05 were identified as differential expression genes (DEGs). As a result, we identified 22,833 DEGs, including 9,509 significant up-regulated genes and 13,324 significant down-regulated genes at stage S2 versus stage S1 (Fig. 4b).

To further understand the biological functions of these DEGs, they were subjected to enrichment analysis of GO terms. In the biological process category, a large number of up-regulation, as well as down-regulation DEGs were enriched in the cellular process, single-organism process and metabolic process. In the cellular component category, most unigenes were classified into cell, cell part and organelle. For the molecular function category; binding and catalytic activity represented the main GO categories (Additional file 5). KEGG analysis showed that 22 pathways were significantly enriched (Fig. 4c). The most represented pathway was carbon metabolism (87, 9.27%), followed by plant hormone signal transduction (76, 8.09%). There were many pathways closely related to seed oil biosynthesis, such as fatty acid metabolism (43, 4.58%), fatty acid biosynthesis (24, 2.56%), unsaturated fatty acid synthesis (24, 2.56%), and glycerolipid metabolism (20, 2.13%), which provide clues for the identification of novel genes involved in TAG synthesis.

Additionally, we found that 312 unigenes encoding TFs were differentially expressed during seed development of *M. oleifera* (Additional file 6). The expression level of 153 TFs were significantly up-regulated, while there were 159 TFs exhibiting obvious down-regulation at stage S2, as compared with stage S1. Interestingly, several TFs critical for seed development and oil accumulation in *Arabidopsis* were identified which were highly expressed at the fast oil accumulation stage (S2), including *WRI1* (Unigene0031370), *ABI3* (Unigene0002005), *FUS3* (Unigene0003221), *ABI5* (Unigene0025890), and *AGL62* (Unigene0034852).

### The unigenes involved into the pathway of triacylglycerol accumulation in *M. oleifera* seeds

Based on KEGG pathway classification and annotation, we identified 198 unigenes involved in fatty acids (FAs) metabolism processes, including FAs de novo biosynthesis in plastid (51 unigenes), elongation (35 uingenes), modification (40 unigenes) and triacylglycerol (TAG) assembly (72 unigenes) in endoplasmic reticulum (see Additional file 7). The expression levels of these unigenes at stages S1 and S2 were summarized Additional file 7. Among these were 66 unigenes with up-regulated expression and 20 unigenes with down-regulated expression at stage S2 as compared with stage S1 (Additional file 7).

**Fig. 5:**
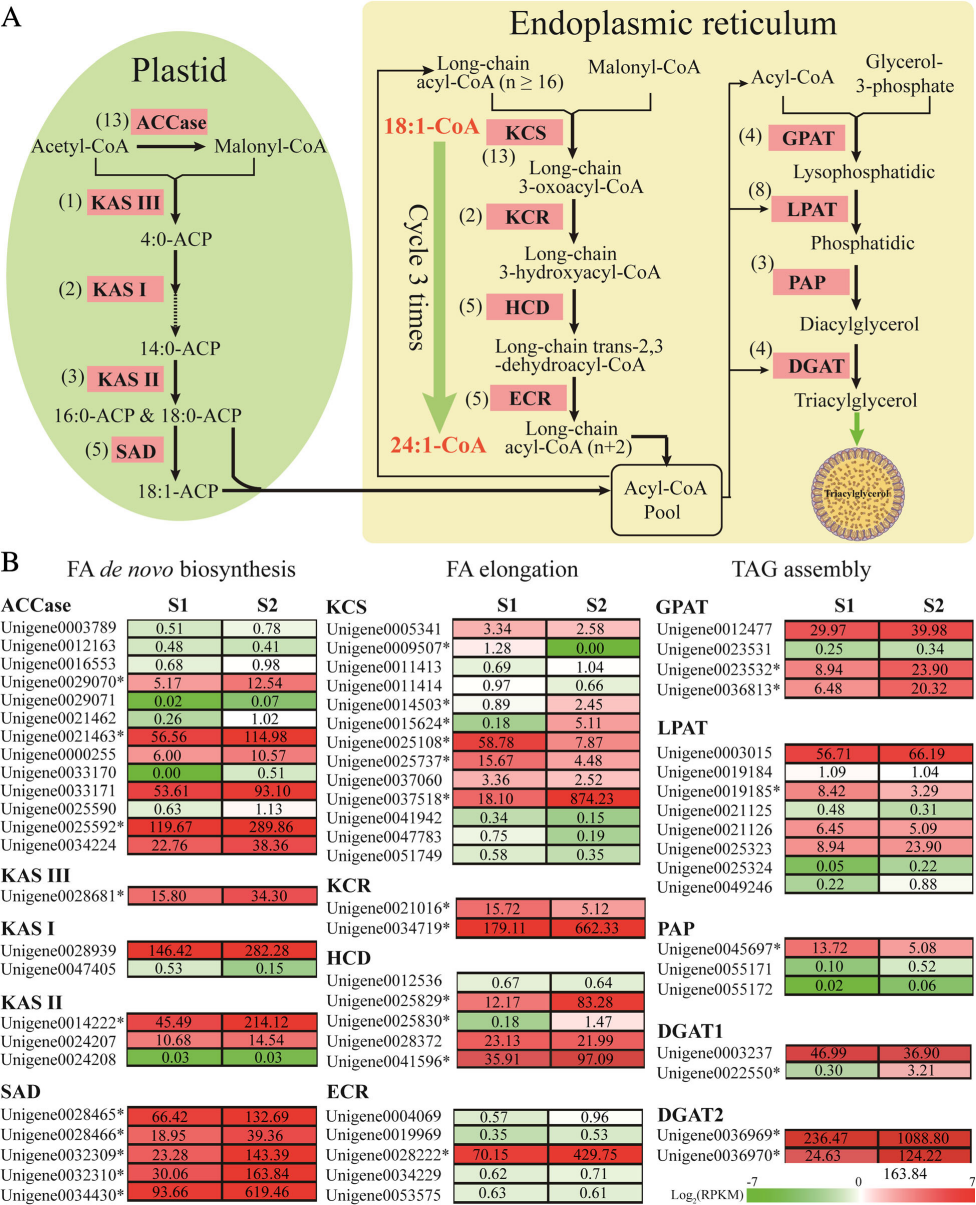
Lipid metabolism and the expression of unigenes in *M oleifera* seeds. **a** Putative unigenes involved in fatty acids and TAG biosynthesis. The number in the brackets indicates the number of unigenes annotated. **b** The heatmap shows the transcript level of each unigene in TAG pathway between the initial stage (S1) and the fast oil accumulation stage (S2). The asterisk indicates the differentially expressed unigenes between two stages (S1 and S2). Number in the colored box represent RPKM values

For fatty acids de novo biosynthesis in plastid, the critical steps and key enzymes were shown in Fig. 5a. Specifically, there were 13 unigenes encoding acetyl-CoA carboxylase (ACCase) subunits (including five biotin carboxylase, three biotin carboxyl carrier proteins, two accA, one accB and two accC), six unigenes encoding fatty acid condensing enzymes (FAS: one for KASIII, two for KASI and three for KASII, respectively), and five unigenes for stearoyl-ACP desaturase (SAD) (Fig. 5a). In this pathway, there were 15 unigenes showing high expression levels for at least one stage of seed development in *M. oleifera*, including six *ACCase*, one *KASIII*, one *KASI*, two *KASII* and five *SAD*. Three *ACCase* genes (Unigene0029070, Unigene0021463, Unigene0025592), one *KASIII* (Unigene0028681) and one *KASII* (Unigene0014222) significantly up-regulated their transcript level at the fast oil accumulation stage. It should be noted that all five *SAD* genes markedly enhanced their transcript level at stage S2 relative to S1 (the initial stage of oil accumulation) (Fig. 5b and Additional file 7).

Our main objective was to identify key genes involved in the biosynthesis of long-chain FAs, especially nervonic acids in endoplasmic reticulum. Here, we fully identified 24 unigenes potentially involved in FAs elongation, including 13 unigenes encoding KCS, two encoding KCR, five encoding HCD and five encoding ECR (Fig. 5a). Further, the full-length cDNA transcript sequences were confirmed by RT-PCR and sequencing for 12 unigenes, including six KCS genes (Unigene0005341, Unigene0014503, Unigene0015624, Unigene0025108, Unigene0025737 and Unigene0037518), three HCD genes (Unigene0025829, Unigene0028372, Unigene0041596), one KCR genes (Unigene0021016) and two ECR genes (Unigene0034229, Unigene0028222). The full-length cDNA transcript sequences of the 12 unigenes were shown in the Table S1 (see Additional file 1). Of 13 *KCS* unigenes, three unigenes (Unigene0009507, Unigene0025108 and Unigene0025737) had a reduced expression level and three unigenes (Unigene0014503, Unigene0015624 and Unigene0037518) up-regulated their expression level during seed development in *M. oleifera*. The *KCS* unigene (Unigene0037518) increased its transcript level at least 48-fold (from 18.1 in S1 to 874.2 in S2) at the fast oil accumulation stage. Of two *KCR* genes, one (Unigene0021016) reduced its expression level, and another (Unigene0034719) significantly increased its expression level (about 3.7-fold) at stage S2. For *HCD* unigenes, Unigene0025830 was expressed at a low level, though its expression increased at stage S2. Two unigenes (Unigene0025829 and Unigene0041596) exhibited high expression level during seed development and was significantly elevated (about 6.9-fold and 2.7-fold, respectively) at stage S2. Only one unigene (Unigene0028222), encoding a ECR enzyme, exhibited a high expression level and about 6-fold transcript increase at the fast oil accumulation stage (Fig. 5b and Additional file 7). Nervonic acids (24:1-CoA) were synthesized via a sequence of four reactions; catalyzing by KCS, KCR, HCD and ECR after three cycles, using the 18:1-CoA as primary substrate (see Fig. 5a). We also found that six unigenes can encode an omega-6 fatty acid desaturase (FAD2) which catalyzes 18C:1 to form 18C:2, and two unigenes encode an omega-3 fatty acid desaturase (FAD3) which further catalyzes 18C:2 to generate 18C:3 (Additional file 7). Four unigenes encoding chloroplast oleate desaturase (FAD6) were identified in *M. oleifera* seeds (Additional file 7).

These FAs (including de novo synthesized, modified or elongated FAs) were subsequently assembled into glycerol-3-phosphate (G-3-P) to form TAG in the ERs which was finally stored in the oil of plant seeds. In this pathway, we found that there were four unigenes encoding GPAT, eight encoding LPAT, three encoding PAP and four unigenes encoding DGAT (including two DGAT1 and two DGAT2); these enzymes perform a critical function in the formation of TAG in the ERs (Fig. 5a). Among 19 unigenes in this pathway, 11 unigenes exhibited a high transcript level in *M. oleifera* seed, and four unigenes substantially up-regulated their expression level including two *GPAT* genes (Unigene0023532 and Unigene0036813), and two *DGAT2* (Unigene0036969 and Unigene0036970) at stage S2 as compared with stage S1 (Fig. 5b and Additional file 7).

### Validation of gene expression using quantitative real-time PCR

**Fig. 6:**
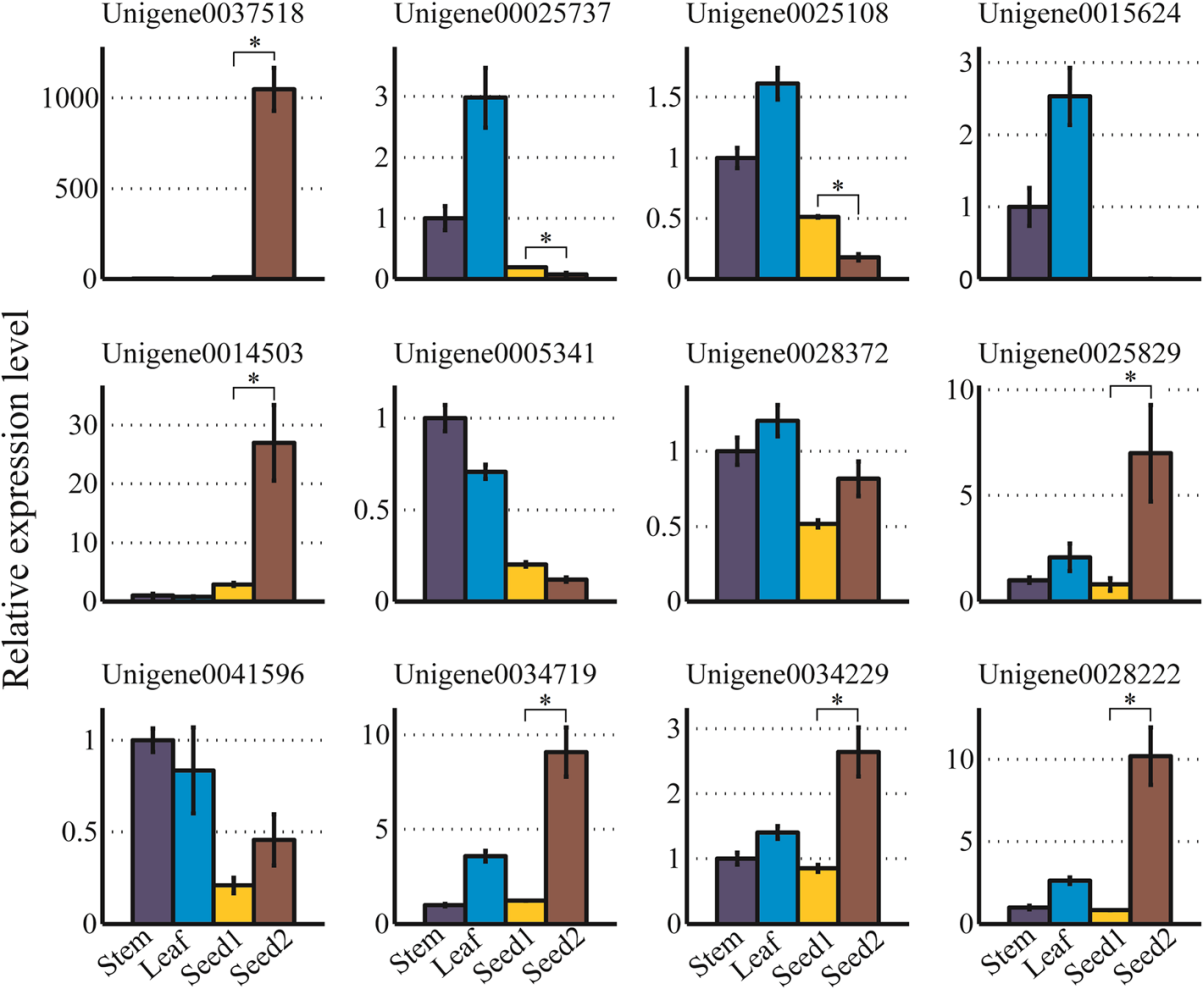
Quantitative real-time PCR analysis of twelve unigenes associated with the fatty acid elongation. Error bars were calculated based on three biological replicates. The expression level of stem sample was normalized to 1. The asterisk above the bars indicates the significant differences between Seed1 and Seed2 (*p* < 0.05)

In order to experimentally validate the expression level of the unigenes involved into biosynthesis of VLCFAs, in particular nervonic acids, 12 unigenes including six *KCS*, three *HCD*, one *KCR* and two *ECR*, were chosen. Quantitative Real-Time PCR (qRT-PCR) was performed on four samples from tender stems, young leaves, and seeds at stages S1 and S2 of development. Overall, the result of qRT-PCR analysis was largely consistent with the transcriptome sequencing data (11 out of the 12 unigenes were detected in the seeds of *M. oleifera*, the exception was Unigene0015624 which had extremely low expression levels in seeds as shown in Fig. 6). For example, four unigenes (Unigene0037518, Unigene0025829, Unigene0034719 and Unigene0028222) were expressed at substantially higher levels at stage S2 relative to stage S1 in the transcriptome sequencing data, which was also confirmed by the qRT-PCR analysis (Fig. 6). The *KCS* gene (Unigene0037518) appeared to be specifically expressed in seeds at the fast oil accumulation stage.

## Discussion

The pathway of fatty acid biosynthesis (including FAs carbon chain elongation and desaturation) is thought to have been conserved in plants. However, the physiological and molecular mechanisms underlying the biosynthesis of unusual FAs, such as nervonic acids in *M. oleifera* and conjugated fatty acids in *Vernicia fordii*, largely remains uncertain. Based on a search for orthologous genes responsible for nervonic acid biosynthesis, we found putative genes that had previously been identified from *Cardamine graeca* [18] and *Lunaria annua* [15] and had their functions analyzed in yeast or *Brassica* oilseeds. Most potential key genes involved in nervonic acid biosynthesis in the FA biosynthesis pathway have yet to be investigated. This study is an important investigation of candidate genes involved in nervonic acid biosynthesis at the transcriptomic level in plant seeds, providing valuable data to improve our understanding of the potential physiological and molecular mechanisms for biosynthesis and accumulation of rich nervonic acid oils in developing *M. oleifera* seeds.

Here, based on high-throughput transcriptome sequencing data we de novo assembled the transcripts in developing seeds of *M. oleifera*, resulting in 55,843 unigenes, that were comparable to transcriptome data from other woody oil-seed plants such as *Vernicia fordii* [41], *Jatropha curcas* [42], and *Camellia oleifera* [43]. Approximately 52.25% of unigenes were annotated and classified into various GO terms or KEGG pathways. The protein homology searches revealed that the transcripts from *M. oleifera* seeds had the highest similarity to transcripts from *V. vinifera*, suggesting that *M. oleifera* is phylogenetically closest to *V. vinifera*. That some transcript sequences (47.75%) had no hits in the public databases might be due to having a shorter sequence length, incomplete protein domain or species-specific sequences in *M. oleifera*. Alternatively, these non-annotated transcripts could be non-coding RNAs such as the precursor of small RNAs or long non-coding RNAs.

One of main objectives of this study was to identify potential unigenes involved in nervonic acid biosynthesis or oil accumulation in developing *M. oleifera* seeds. Nervonic acid was synthesized in ER by using oleic acid (18C:1^Δ9^) as the substrate, then catalyzed by FAE complex composed of KCS, KCR, HCD and ECR [14, 17, 20]. The oleic acid content (18C:1) was high (over 32%) and relatively stable during seed development (from stages S1 to S3) until seed maturity (stage S4) as shown in Additional file 2. This quantity of oleic acid appears to be sufficient to act as a substrate for nervonic acid biosynthesis. Throughout seed development there were high levels of expression of all five *SAD* genes that are responsible for producing 18C:1 from the substrate 18C:0 (see Fig. 5b). This may account for the relatively high content of 18C:1 that was maintained at the transcription level in developing seeds of *M. oleifera*. At the fast oil accumulation stage (S2), the proportion of nervonic acid increased rapidly (from 0.88 to 29.39%), suggesting that FAs elongation began during oil accumulation at stage S2. There has been increasing evidence that KCS is the rate-limiting enzyme in fatty acid elongation and that its expression level is an importance determinant of the final VLCFAs content [27, 28, 44, 45]. The heterologous expression of *KCS* cloned from *Cardamine graeca* or *Lunaria annua* can produce or increase nervonic acid content in transgenic cruciferous plants [15, 18]. In current study, we identified 13 putative *KCS* genes in *M. oleifera* seeds, but have yet to determine the functions of these genes. In particular, the *KCS* unigene (Unigene0037518) exhibited strongly seed-specific expression with an at least 48-fold increase at the transcript level at the fast oil accumulation stage, which strongly indicates that this gene could drive nervonic acid biosynthesis in *M. oleifera* seeds. Further functional characterization of Unigene0037518 and its substrate specificity assay are required to determine whether it is species-specific; encoding the rate-limiting enzyme for catalyzing nervonic acids in *M. oleifera* seeds.

Also, several unigenes encoding KCR, HCD and ECR enzymes, which are part of fatty acid carbon-chain elongation in the pathway of FA biosynthesis, were identified. They probably contribute to the accumulation of nervonic acid oils in *M. oleifera* seeds. Thus, these targeted genes could be important sources for genetic and metabolic engineering for obtaining nervonic acid production by heterologous transgenic technique.

The rich nervonic acids incorporated in TAG molecules are usually dependent not only on the efficient synthesis of nervonic acids, but on the efficient assembly system for the selective or specific incorporation of nervonic acids into TAG. Generally, the *DGAT* genes are thought to play a critical role in catalyzing the final step of triacylglycerol (TAG) biosynthesis in developing oleaginous seeds [46]. Different types of *DGAT*s, such as *DGAT1* and *DGAT2*, usually exhibit structural and functional divergence [47], thus *DGAT1* has been thought to be responsible for regulating or controlling oil content, whereas *DGAT2* was thought to be responsible for selectively or specifically incorporating specific fatty acids into TAG in plants [35, 46, 48]. Here, two homologous *DGAT2* genes identified in *M. oleifera* exhibited a high level of expression in developing seeds; strongly implying that these two genes could play a critical role in selectively or specifically incorporating nervonic acids into TAG in *M. oleifera* seeds. If so, these two *DGAT2s* could be combined with the targeted nervonic acid biosynthesis genes identified in this study by genetic engineering to enhance the nervonic acids content in TAGs. In sum, we identified several candidate genes involved in the nervonic acids biosynthesis and TAG assembly, but the molecular basis of high-efficient biosynthesis of nervonic acids in *M. oleifera* seeds remains to be elucidated. Such study is required to determine whether these candidate genes are unique to *M. oleifera*, as well as, deduce whether nervonic acids biosynthesis in *M. oleifera* seeds is closely correlated to the strong seed-specific expression of these identified genes or to the sequence variation when compared with homologous genes from other species, which may alter the enzyme activity or substrate specificity.

We identified a large number of TFs, which were highly expressed at the fast oil accumulation stage of developing seeds. Most of these TFs are functionally uncharacterized or unknown. However, we detected some transcriptional regulators such as WRI1, ABI3 and FUS3, which are expressed in a seed-specific manner and documented to be functionally involved in regulation of lipid biosynthesis in seed development of *Arabidopsis* and other plants [49]. For example, loss-of-function in mutant of *AtWRI1* substantially reduced the seed oil content compared to the wild-type in Arabidopsis [50]. Overexpression of *WRI1* significantly enhanced the seed oil content in transgenic plants [51]. Increasing evidence has showed that *WRI1* is a master regulator in controlling the gene expression of lipid genes in the pathway of fatty acid biosynthesis [52, 53]. Studies have also revealed that both ABI3 and FUS3 are involved in direct or indirect regulation of the fatty acid biosynthesis and TAG accumulation in other plants [54, 55]. Interestingly, the VLCFAs content in the *abi3* and *fus3* mutant seeds was significantly decreased, which is associated with reduced activity of FAE1 (a key fatty acid carbon-chain elongase that regulates production of VLCFAs) [56]. Probably, the identified WRI1, ABI3 and FUS3 are involved in the regulation of the biosynthesis processes of rich nervonic acids oils in *M. oleifera* seeds.

## Conclusion

The current study comprehensively reported transcriptome data from nervonic acid oil producing *M. oleifera* seeds, and identified genes that are potentially critical for driving the processes of nervonic acid biosynthesis and TAG assembly. These results contribute to our understanding of the potential physiological and molecular mechanisms of biosynthesis and accumulation of rich nervonic acid oils in developing *M. oleifera* seeds. The study has also produced targeted gene resources that can be used for genetic and metabolic engineering for future biotechnological approaches to nervonic acid production.

## Additional files

Additional file 1: **Table S1.** Primers used in this study for quantitative real-time PCR. (DOC 26 kb)

Additional file 2: **Table S2.** Fatty acid (FA) composition of oil extracted from seeds at different development stages in *Malania oleifera*. (DOC 24 kb)

Additional file 3: **Figure S1.** E-value distribution of best BLAST hits for each unigene with a cutoff E-value of 1.0E-5 from different databases. (DOC 119 kb)

Additional file 4: **Table S3**. The KEGG classification of unigenes identified in *Malania oleifera*. (XLS 41 kb)

Additional file 5: **Figure S2.** Gene Ontology (GO) enrichment analysis of differentially expressed unigenes during seed development. (DOC 122 kb)

Additional file 6: **Table S4.** Differentially expressed transcriptor factors during seed development of *Malania oleifera*. (XLS 37 kb)

Additional file 7: **Table S5.** Unigenes involved into the pathway of fatty acids synthesis, modification, elongation and triacylglycerol assembly in *Malania oleifera* seeds. (XLS 71 kb)

## References

[1] Yang LH, Ding KY, Lu SG (2003). The karyotype of *Malania oleifera*. Acta Bot Yunnanica.

[2] Li SG (1980). *Malania*, a new genus of oil-yielding plant. Bull Bot Lab North-East Forest Inst.

[3] Qiu XH, Lin YR (1988). Flora of China.

[4] Fu LG (1992). Red data book of Chinese plant-the rare and endangered plants.

[5] Poulos A (1995). Very long chain fatty acids in higher animals-a review. Lipids.

[6] Merrill AH, Schmelz EM, Wang E, Dillehay DL, Rice LG, Meridith F (1997). Importance of sphingolipids and inhibitors of sphingolipid metabolism as components of animal diets. J Nutr.

[7] Farquharson J, Jamieson EC, Abbasi KA, Parrick WJ, Logan RW, Cockburn F (1995). Effect of diet on the fatty acid composition of the major phospholipids of infant cerebral cortex. Arch Dis Child.

[8] Assies J, Lieverse R, Vreken P, Wanders RJ, Dingemans PM, Linszen DH (2001). Significantly reduced docosahexaenoic and docosapentaenoic acid concentrations in erythrocyte membranes from schizophrenic patients compared with a carefully matched control group. Biol Psychiatry.

[9] Evans DA, Bennett DA, Wilson RS, Bienias JL, Morris MC, Scherr PA (2003). Incidence Alzheimer disease in a biracial urban community: relation to apolipoprotein E allele status. Arch Neurol.

[10] Chen JR, Hsu SF, Hsu CD, Hwang LH, Yang SC (2004). Dietary patterns and blood fatty acid composition in children with attention-deficit hyperactivity disorder in Taiwan. J Nutr Biochem.

[11] Pamplona R, Dalfó E, Ayala V, Bellmunt MJ, Prat J, Ferrer I (2005). Proteins in human brain cortex are modified by oxidation, glycoxidation, and lipoxidation: effects of Alzheimer disease and identification of lipoxidation targets. J Biol Chem.

[12] Tanaka K, Shimizu T, Ohtsuka Y, Yamashiro Y, Oshida K (2007). Early dietary treatments with Lorenzo’s oil and docosahexaenoic acid for neurological development in a case with Zellweger syndrome. Brain Dev.

[13] Amminger GP, Schäfer MR, Klier CM, Slavik JM, Holzer I, Holub M (2012). Decreased nervonic acid levels in erythrocyte membranes predict psychosis in help-seeking ultra-high-risk individuals. Mol Psychiatry.

[14] Kasai N, Mizushina Y, Sugawara F, Sakaguchi K (2002). Three-dimensional structural model analysis of the binding site of an inhibitor, nervonic acid, of both DNA polymerase beta and HIV-1 reverse transcriptase. J Biochem.

[15] Guo Y, Mietkiewska E, Francis T, Katavic V, Brost JM, Giblin M (2009). Increase in nervonic acid content in transformed yeast and transgenic plants by introduction of a *Lunaria annua* L. *3-ketoacyl-CoA synthase* (*KCS*) gene. Plant Mol Biol.

[16] Wang XY, Wang SQ (2005). A new resource of nervonic acid: purpleblow maple oil. China Oils Fats.

[17] Bettger WJ, McCorquodale ML, Blackadar CB (2001). The effect of a *Tropaeolum speciosum* oil supplement on the nervonic acid content of sphingomyelin in rat tissues. J Nutr Biochem.

[18] Taylor DC, Francis T, Guo Y, Brost JM, Katavic V, Mietkiewska E (2009). Molecular cloning and characterization of a *KCS* gene from *Cardamine graeca* and its heterologous expression in *Bracssica* oilseeds to engineer high nervonic acid oils for potential medical and industrial ues. Plant Biotechnol J.

[19] Ohlrogge J, Browse G (1995). Lipid biosynthesis. Plant Cell.

[20] Ohlrogge JB, Jaworski JG (1997). Regulation of fatty acid synthesis. Annu Rev Plant Physiol Plant Mol Biol.

[21] Bach L, Faure JD (2010). Role of very-long-chain fatty acids in plant development, when chain length does matter. C R Biol.

[22] Kunst L, Samuels L (2009). Plant cuticles shine: advances in wax biosynthesis and export. Curr Opin Plant Biol.

[23] Kunst L, Taylor DC, Underhill EW (1992). Fatty acid elongation in developing seeds of *Arabidopsis thaliana*. Plant Physiol Biochem.

[24] Franke R, Höfer R, Briesen I, Emsermann M, Efremova N, Yephremov A (2009). The *DAISY* gene from *Arabidopsis* encodes a fatty acid elongase condensing enzyme involved in the biosynthesis of aliphatic suberin in roots and the chalaza-micropyle region of seeds. Plant J.

[25] Todd J, Post-Beittenmiller D, Jaworski JG (1999). *KCS1* encodes a fatty acid elongase 3-ketoacyl-CoA synthase affecting wax biosynthesis in *Arabidopsis thaliana*. Plant J.

[26] Millar AA, Kunst L (1997). Very-long-chain fatty acid biosynthesis is controlled through the expression and specificity of the condensing enzyme. Plant J.

[27] Lassner MW, Lardizabal K, Metz JG (1996). A jojoba beta-Ketoacyl-CoA synthase cDNA complements the canola fatty acid elongation mutation in transgenic plants. Plant Cell.

[28] James DW, Lim E, Keller J, Plooy I, Ralston E, Dooner HK (1995). Directed tagging of the Arabidopsis *FATTY ACID ELONGATION1* (*FAE1*) gene with the maize transposon *activator*. Plant Cell.

[29] Gan L, Wang X, Cheng Z, Liu L, Wang J, Zhang Z (2016). *Wax crystal-sparse leaf 3* encoding a β-ketoacyl-CoA reductase is involved in cuticular wax biosynthesis in rice. Plant Cell Rep.

[30] Beaudoin F, Wu X, Li F, Haslam RP, Markham JE, Zheng H (2009). Functional characterization of the Arabidopsis *β*-ketoacyl-coenzyme a reductase candidates of the fatty acid elongase. Plant Physiol.

[31] Bach L, Michaelson LV, Haslam R, Bellec Y, Gissot L, Marion J (2008). The very-long-chain hydroxy fatty acyl-CoA dehydratase PASTICCINO2 is essential and limiting for plant development. Proc Natl Acad Sci U S A.

[32] Zheng H, Rowland O, Kunst L (2005). Disruptions of the Arabidopsis Enoyl-CoA reductase gene reveal an essential role for very-long-chain fatty acid synthesis in cell expansion during plant morphogenesis. Plant Cell.

[33] Xu R, Wang R, Liu A (2011). Expression profiles of genes involved in fatty acid and triacylglycerol synthesis in developing seeds of Jatropha (*Jatropha curcas* L.). Biomass Bioenergy.

[34] Baud S, Lepiniec L (2010). Physiological and developmental regulation of seed oil production. Prog Lipid Res.

[35] Xu R, Yang T, Wang R, Liu A (2014). Characterization of *DGAT1* and *DGAT2* from *Jatropha curcas* and their functions in storage lipid biosynthesis. Funct Plant Biol.

[36] Yang Tianquan, Xu Ronghua, Chen Jianghua, Liu Aizhong (2016). β-Ketoacyl-acyl Carrier Protein Synthase I (KASI) Plays Crucial Roles in the Plant Growth and Fatty Acids Synthesis in Tobacco. International Journal of Molecular Sciences.

[37] Grabherr MG, Haas BJ, Yassour M, Levin JZ, Thompson DA, Amit I (2011). Full-length transcriptome assembly from RNA-seq data without a reference genome. Nat Biotechnol.

[38] Conesa A, Götz S, García-Gómez JM, Terol J, Talón M, Robles M (2005). Blast2GO: a universal tool for annotation, visualization and analysis in functional genomics research. Bioinformatics.

[39] Ye J, Fang L, Zheng H, Zhang Y, Chen J, Zhang Z (2006). WEGO: a web tool for plotting GO annotations. Nucleic Acids Res.

[40] Mortazavi A, Williams BA, McCue K, Schaeffer L, Wold B (2008). Mapping and quantifying mammalian transcriptomes by RNA-Seq. Nat Methods.

[41] Galli V, Guzman F, Messias RS, Körbes AP, Silva SD, Margis-Pinheiro M (2014). Transcriptome of tung tree mature seeds with an emphasis on lipid metabolism genes. Tree Genet Genomes.

[42] Costa GG, Cardoso KC, Del Bem LE, Lima AC, Cunha MA, de Campos-Leite L (2010). Transcriptome analysis of the oil-rich seed of the bioenergy crop Jatropha curcas L. BMC Genomics.

[43] Xia EH, Jiang JJ, Huang H, Zhang LP, Zhang HB, Gao LZ (2014). Transcriptome analysis of the oil-rich tea plant, *Camellia oleifera*, reveals candidate genes related to lipid metabolism. PLoS One.

[44] Huai D, Zhang Y, Zhang C, Cahoon EB, Zhou Y (2015). Combinatorial effects of fatty acid elongase enzymes on nervonic acid production in *Camelina sativa*. PLoS One.

[45] Mietkiewska E, Brost JM, Giblin EM, Barton DL, Taylor DC (2007). Cloning and functional characterization of the fatty acid elongase 1 (FAE1) gene from high erucic *Crambe abyssinica* cv. Prophet Plant Biotechnol J.

[46] Shockey JM, Gidda SK, Chapital DC, Kuan JC, Dhanoa PK, Bland JM (2006). Tung tree DGAT1 and DGAT2 have nonredundant functions in triacylglycerol biosynthesis and are localized to different subdomains of the endoplasmicreticulum. Plant Cell.

[47] Turchetto-Zolet AC, Maraschin FS, de Morais GL, Cagliari A, Andrade CM, Margis-Pinheiro M (2011). Evolutionary view of acyl-CoA diacylglycerol acyltransferase (DGAT), a key enzyme in neutral lipid biosynthesis. BMC Evol Biol.

[48] Burgal J, Shockey J, Lu C, Dyer J, Larson T, Graham I (2008). Metabolic engineering of hydroxy fatty acid production in plants: RcDGAT2 drives dramatic increases in ricinoleate levels in seed oil. Plant Biotechnol J.

[49] Le BH, Cheng C, Bui AQ, Wagmaister JA, Henry KF, Pelletier J (2010). Global analysis of gene activity during Arabidopsis seed development and identification of seed-specific transcription factors. Proc Natl Acad Sci U S A.

[50] Focks N, Benning C (1998). Wrinkled1: a novel, low-seed-oil mutant of Arabidopsis with a deficiency in the seed-specific regulation of carbohydrate metabolism. Plant Physiol.

[51] Kong Q, Ma W (2018). WRINKLED1 transcription factor: how much do we know about its regulatory mechanism?. Plant Sci.

[52] Baud S, Mendoza MS, Harscoët E, Lepiniec L, Dubreucq B, To A (2007). WRINKLED1 specifies the regulatory action of LEAFY COTYLEDON2 towards fatty acid metabolism during seed maturation in Arabidopsis. Plant J.

[53] Maeo K, Tokuda T, Ayame A, Mitsui N, Kawai T, Tsukagoshi H (2009). An AP2-type transcription factor, WRINKLED1, of *Arabidopsis thaliana* binds to the AW-box sequence conserved among proximal upstream regions of genes involved in fatty acid synthesis. Plant J.

[54] Stone SL, Braybrook SA, Paula SL, Kwong LW, Meuser J, Pelletier J (2008). Arabidopsis LEAFY COTYLEDON2 induces maturation traits and auxin activity: implications for somatic embryogenesis. Proc Natl Acad Sci U S A.

[55] Chiu RS, Nahal H, Provart NJ, Gazzarrini S (2012). The role of the Arabidopsis FUSCA3 transcription factor during inhibition of seed germination at high temperature. BMC Plant Biol.

[56] Roscoe TT, Guilleminot J, Bessoule JJ, Berger F, Devic M (2015). Complementation of seed maturation phenotypes by ectopic expression of ABSCISIC ACID INSENSITIVE3, FUSCA3 and LEAFY COTYLEDON2 in Arabidopsis. Plant Cell Physiol.

