# Supplementary material for: Transcriptome analysis reveals crucial genes involved in the biosynthesis of nervonic acid in woody Malania oleifera oilseeds
Source: BMC Plant Biol. 2018 Oct 19;18:247. doi: 10.1186/s12870-018-1463-6 (PMC6195686; doi:10.1186/s12870-018-1463-6)
Supplement: Supplementary file 1 — Table S1. Primers used in this study for quantitative real-time PCR. (DOC 26 kb) [file 12870_2018_1463_MOESM1_ESM.doc]

Table S1. Primers used in this study for quantitative real-time PCR.

| Unigenes | Sequences |
| --- | --- |
| Q0037518-F | CCTACCACCTCTTGCAGTCA |
| Q0037518-R | AGAAAGACAAGGGTGCTGGA |
| Q0025737-F | TACATGATGACCAGGCCTCG |
| Q0025737-R | AGTGCTCCATGAATCGGTGA |
| Q0025108-F | TCCTTTCCCCTCTTGTGGTC |
| Q0025108-R | ACAAGGTATACAGGGCGAGG |
| Q0014503-F | GTGGGAAGTGGTAAGGGACA |
| Q0014503-R | GGTCATTGATCCACCCATGC |
| Q0005341-F | TCCTCCAGAGCGTCAATCTC |
| Q0005341-R | TCGTGGGGATTCATTTGGGA |
| MoUB-F | CTCTGCAACCTCCTCAAGTC |
| MoUB-R | TTGTGTACATCCTCGCCATG |
| Q0015624-F | TTCGTCTTCACCCTCTCAGT |
| Q0015624-R | CGACGACTCCAGTATCCTCT |
| Q0034719-F | ATGCGAAGTTCTTCCACGAA |
| Q0034719-R | CAGAGGGGATCACAATTGCA |
| Q0028372-F | GCAGCTTCGTATCCATCCAT |
| Q0028372-R | GAAAATGAGTCCTCCCACCC |
| Q0025829-F | GATACGGTTCGCTGGTCTTT |
| Q0025829-R | CCAAATGCCTCTTTCATGCC |
| Q0041596-F | TGAAGATGGGGATCGTGGAT |
| Q0041596-R | CAATCCCAGCACGACACATA |
| Q0034229-F | GAGGCATGATCTTGGACTCC |
| Q0034229-R | CTTCAAGTCAATGGGAGGGG |
| Q0028222-F | CCACCCGGTTCAGACATATG |
| Q0028222-R | TTTGAGAGGGGTGAAGTTGC |
