# Supplementary material for: Transcriptome analysis reveals crucial genes involved in the biosynthesis of nervonic acid in woody Malania oleifera oilseeds
Source: BMC Plant Biol. 2018 Oct 19;18:247. doi: 10.1186/s12870-018-1463-6 (PMC6195686; doi:10.1186/s12870-018-1463-6)
Supplement: Supplementary file 2 — Table S2. Fatty acid (FA) composition of oil extracted from seeds at different development stages in Malania oleifera. (DOC 24 kb) [file 12870_2018_1463_MOESM2_ESM.doc]

Table S2. Fatty acid (FA) composition of oil extracted from seeds at different development stages in *Malania oleifera*.

| FA species | S1 | S2 | S3 | S4 |
| --- | --- | --- | --- | --- |
| C16:0 | 13.87% | 2.45% | 0.90% | 0.98% |
| C16:1 | 0.22% | 0.15% | 0.29% | 0.18% |
| C18:0 | 1.51% | 0.55% | 0.35% | 0.39% |
| C18:1 | 37.64% | 38.09% | 32.66% | 3.52% |
| C18:2 | 30.96% | 5.32% | 2.42% | 1.45% |
| C18:3 | 8.87% | 2.24% | 1.00% | 0.68% |
| C20:0 | 0.98% | 0.61% | 0.51% | 2.90% |
| C20:1 | 1.17% | 3.09% | 2.70% | 0.14% |
| C22:0 | 0.62% | 1.54% | 1.38% | 1.77% |
| C22:1 | 0.90% | 14.88% | 14.76% | 21.33% |
| C24:0 | 2.38% | 1.69% | 1.93% | 2.87% |
| C24:1 | 0.88% | 29.39% | 41.10% | 63.79% |

S1: initial stage; S2: fast oil accumulation stage; S3: one month later than S2; S4: maturing seed.
