# Supplementary material for: Transcriptome analysis reveals crucial genes involved in the biosynthesis of nervonic acid in woody Malania oleifera oilseeds
Source: BMC Plant Biol. 2018 Oct 19;18:247. doi: 10.1186/s12870-018-1463-6 (PMC6195686; doi:10.1186/s12870-018-1463-6)
Supplement: Supplementary file 3 — Figure S1. E-value distribution of best BLAST hits for each unigene with a cutoff E-value of 1.0E-5 from different databases. (DOC 119 kb) [file 12870_2018_1463_MOESM3_ESM.doc]

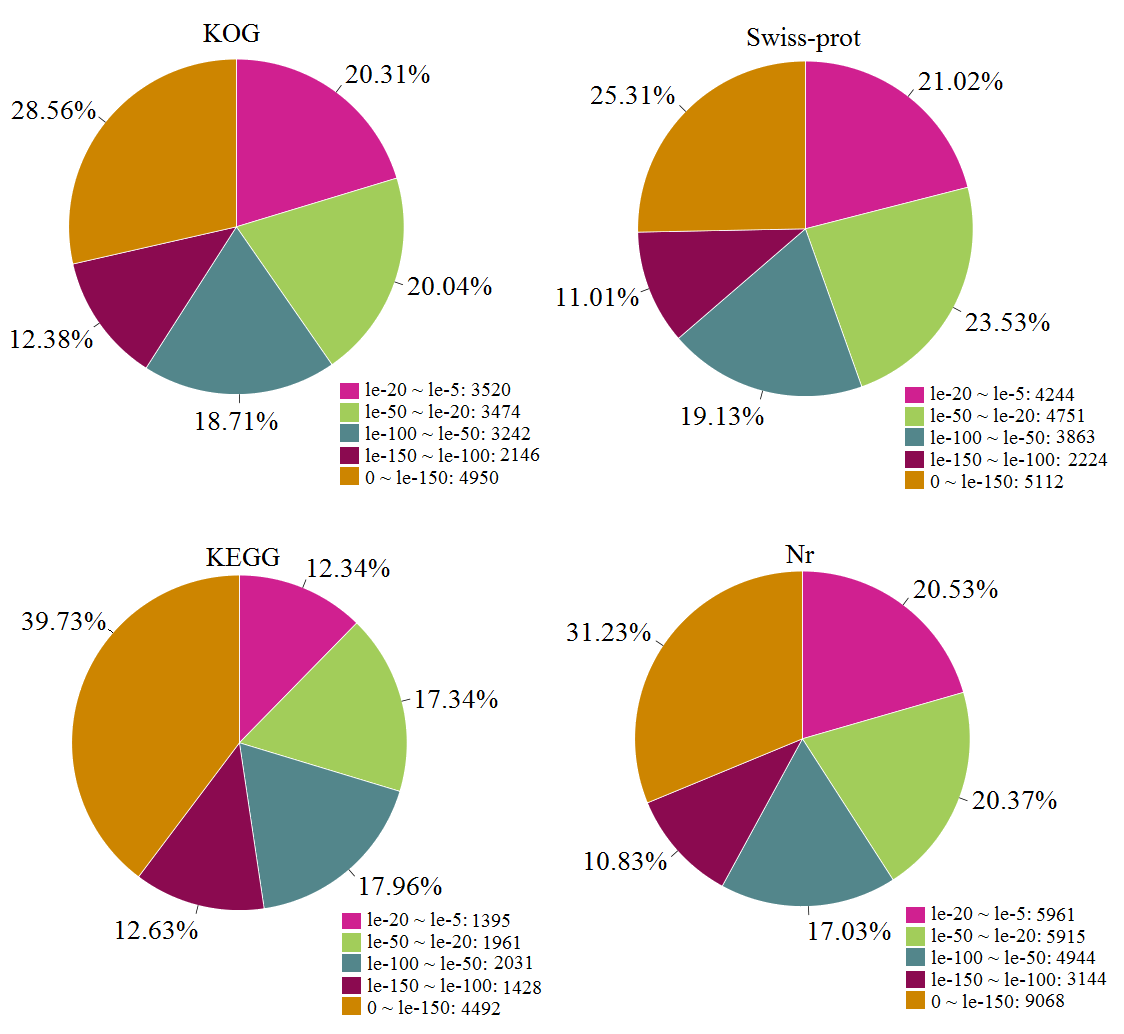


**Figure S1.** E-value distribution of best BLAST hits for each unigene with a cutoff E-value of 1.0E-5 from different databases.
