# Supplementary material for: Transcriptome analysis reveals crucial genes involved in the biosynthesis of nervonic acid in woody Malania oleifera oilseeds
Source: BMC Plant Biol. 2018 Oct 19;18:247. doi: 10.1186/s12870-018-1463-6 (PMC6195686; doi:10.1186/s12870-018-1463-6)
Supplement: Supplementary file 5 — Figure S2. Gene Ontology (GO) enrichment analysis of differentially expressed unigenes during seed development. (DOC 122 kb) [file 12870_2018_1463_MOESM5_ESM.doc]

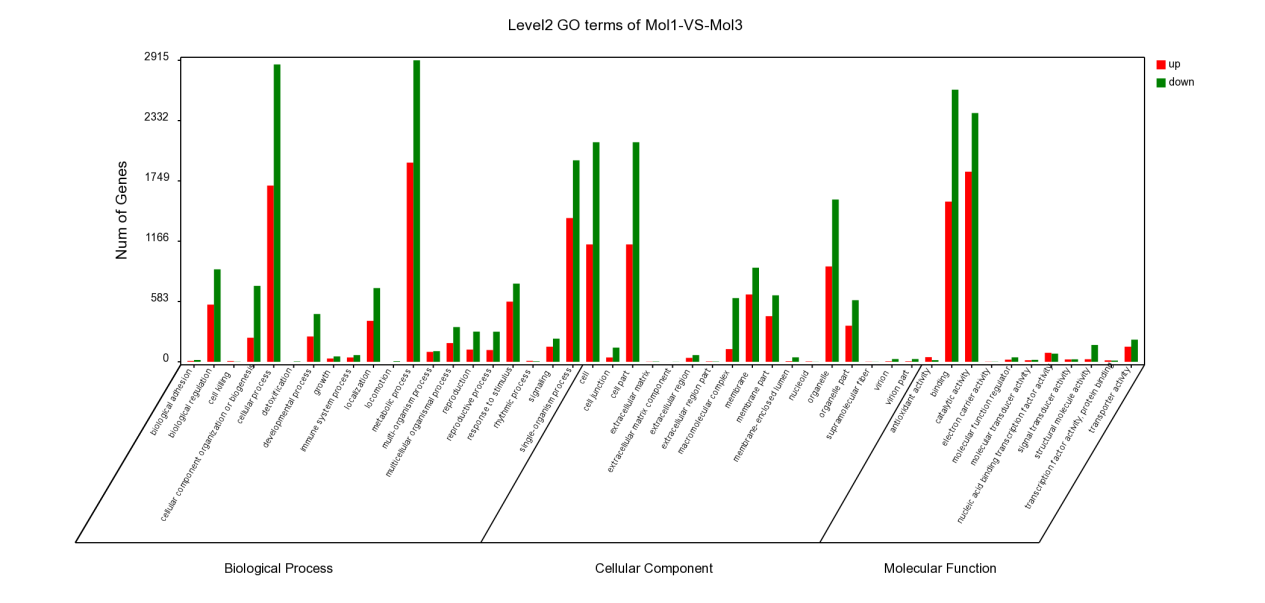


**Figure S2.** Gene Ontology (GO) enrichment analysis of differentially expressed unigenes during seed development.
